# Supplementary material for: A framework to measure transit-oriented development around transit nodes: Case study of a mass rapid transit system in Dhaka, Bangladesh
Source: PLoS One. 2023 Jan 6;18(1):e0280275. doi: 10.1371/journal.pone.0280275 (PMC9821780; doi:10.1371/journal.pone.0280275)
Supplement: S1 Table — (DOCX) [file pone.0280275.s001.docx]

**Table A: Indicator weights with criteria**

| **Criteria** | **Indicators** | **Indicator Weights** |
| --- | --- | --- |
| Density | Population density | 0.5 |
|  | Commercial density | 0.33 |
|  | Employment density | 0.17 |
| Diversity of Land Use | Land use diversity (Mix Percent) | 1.0 |
| Destination accessibility | Land use mixedness | 0.17 |
|  | Length of walkable/cyclable paths | 0.5 |
|  | Intersection density | 0.33 |
| Design | Parking utilization | 0.5 |
|  | Open/green spaces | 0.5 |

Source: Partially adapted from Singh et al. (2015) [55]
